# Double Internal Limiting Membrane Insertion for Macular Hole-Associated Retinal Detachment

**DOI:** 10.1155/2017/3236516

**Published:** 2017-07-30

**Authors:** San-Ni Chen, Chung-May Yang

**Affiliations:** ^1^Department of Ophthalmology, Changhua Christian Hospital, Changhua City, Taiwan; ^2^College of Medicine, Chung-Shan Medical University, Taichung, Taiwan; ^3^Department of Ophthalmology, National Taiwan University Hospital, Taipei, Taiwan; ^4^College of Medicine, National Taiwan University, Taipei, Taiwan

## Abstract

**Purpose:**

To describe a modified technique of internal limiting membrane (ILM) insertion for macular hole- (MH-) associated retinal detachment (RD) in highly myopic eyes.

**Methods:**

Nine eyes underwent pars plana vitrectomy, cortical vitreous removal, and fovea-sparing ILM peeling. Double ILM insertion into the hole was performed with inverted perifoveal ILM and a free ILM flap followed by air-fluid exchange.

**Results:**

Two of the 9 eyes had perifoveal ILM partially torn after cortical vitreous or epiretinal removal. All eyes had the ILM plug stabilized within the MH after double ILM insertion. Postoperatively, MH was sealed with the retina reattached in all the eyes.

**Conclusion:**

Double ILM insertion may further secure the ILM flap in place in the eyes with MH-associated RD, especially in cases in which insufficient perifoveal ILM was left. This trial is registered with the clinical registration number Clinicaltrials.gov NCT03174639.

## 1. Introduction

Macular hole- (MH-) associated retinal detachment (RD) in high myopia poses a specific challenge for the vitreoretinal surgeons. Although the anatomical reattachment rate is high after modern techniques of vitrectomy and membrane peeling were performed, the hole closure rate has been low, usually around 50 percent [1–3]. An open MH not only compromises central vision but also runs the risk of recurrent RD [2, 3]. Recently, inverted internal limiting membrane (ILM) flap covering the hole has been advocated to bridge the hole and facilitate hole closure [4–8]. Although inverted ILM flap technique may increase the hole closure rate, the natural tendency of the ILM to flip back may potentially prevent an adequate bridging effect postoperatively. Covering the ILM flap with a layer of blood has been proposed recently to stabilize the ILM flap [5]. Recently, we proposed another technique by inserting the inverted perifoveal ILM about 1.5 disc diameter in size into the hole to facilitate hole closure. In 20 cases treated with this technique, we were able to achieve a 100% closure rate in highly myopic eyes with MH-associated RD [6]. However, during the insertion procedure, prolonged manipulation may sometimes be required because the ILM tissue tends to fold back. In addition, part of the parafoveal ILM tissue may sometimes be torn away during cortical vitreous stripping or epiretinal membrane (ERM) removal, leaving insufficient perifoveal ILM tissues for a proper insertion. Thus, we have modified the inverted ILM flap technique [6] by adding another piece of free ILM flap into the hole to address the abovementioned problems. We found that this additional step made the plugging of the ILM tissue faster and the resultant ILM plug much more secure. We therefore reported a small case series using this modified inverted ILM flap insertion technique; details of the surgical techniques were described.

## 2. Material and Methods

This is a retrospective study. From July 2015 to December 2015, clinical records of 9 consecutive cases of MH with RD in high myopia treated with combined inverted and free ILM flap insertion into the hole were reviewed. All patients had informed consent. None of the 9 cases had a history of trauma or concurrent peripheral breaks. Two experienced surgeons (SN Chen and CM Yang from Changhua Christen Hospital and National Taiwan University Hospital, resp.) performed the operation. The study was approved by the ethics committee and research board of the two hospitals.

High myopia was defined as an eye with a refractive error of more than six diopters or an axial length of more than 26 mm. Staphyloma was defined as ectasia in the posterior pole detected in the OCT and fundus photographs. The severity of RD was separated into two types: within the equator or beyond the equator.

Each patient underwent thorough ophthalmological examinations before and after surgery. All cases were followed up for more than 3 months after surgery.

## 3. Surgical Technique

**Figure 1:**
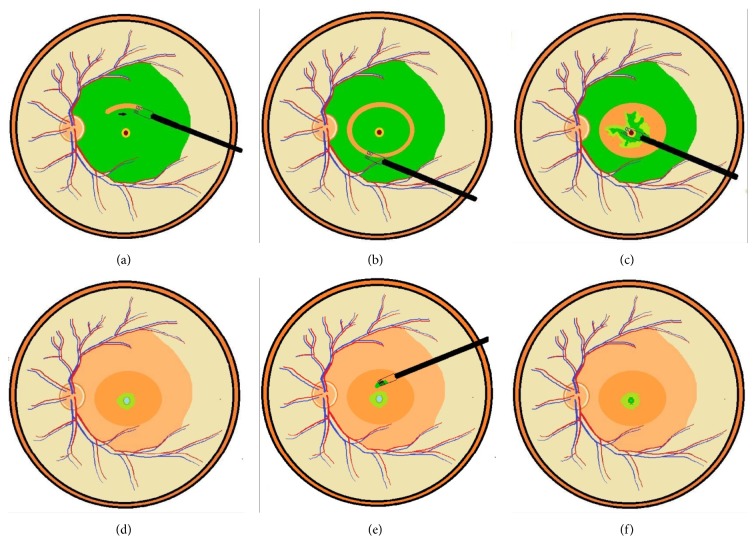
Direct forceps grasping was used to create an ILM break where a good ICG staining had been obtained (a). A ring-shaped ILM flap was created around the macular hole (b). The ILM was detached from the retina to the edge of the macular hole (c). Further anterior ILM peeling was performed up to the arcade along with the overlying ERM. The ILM flap anchoring on the hole edge was inverted and inserted into the hole using microforceps (d). A small amount of Viscoat was then carefully applied on top of the hole. After the infusion was turned off, a piece of the previously obtained free ILM flap was released from the microforceps on top of the macular hole (e). The microforceps with closed tips was used to guide the ILM tissue to fall on the hole and to nudge the free ILM tissue into the hole (f).

Standard 3-port 23-gauged pars plana vitrectomy was performed. After core vitrectomy, triamcinolone acetonide-assisted (TA, 2.5 mg/ml) posterior hyaloid removal followed by forceps removal of any ERM was performed. A 24-gauge blunt-tipped needle connected with Viscoat® (Alcon Laboratories, Fort Worth, TX, USA) containing a syringe was placed within the hole just below the level of the macular hole. A small amount of Viscoat was injected into and around the hole. An ICG solution (25 mg ICG in 15 ml 5% glucose-water solution, final concentration = 1.7 mg/ml) was then carefully applied around the macular hole within the arcade. Excessive ICG was immediately removed by suction. Direct forceps grasping was then used to create an ILM break where a good ICG staining had been obtained (Figure 1(a)). ILM at the parafoveal area was peeled in a circular fashion. Care was taken not to peel the ILM flap across the hole edge. The pieces of ILM flap removed were saved on a balance salt-solution-soaked sponge tissue for later use. At least 1.5 to 2 disc areas of the ILM around the hole were left in place (Figure 1(b)) but was detached from the retina up to the edge of the hole (Figure 1(c)). Excessive ILM was carefully trimmed with the vitreous cutter with low suction in a shaving mode. Further anterior ILM peeling was performed up to the arcade along with the overlying ERM. The peripheral retina was then examined for possible breaks. The ILM flap anchoring on the hole edge was inverted and inserted into the hole using microforceps. All free-floating ends of the ILM tissue were carefully trapped under the hole (Figure 1(d)). Intraocular pressure was kept low to minimize turbulence. A small amount of Viscoat was then carefully applied on top of the hole. A piece of previously obtained free ILM flap about the size of the hole was picked up with microforceps and was released in the mid-vitreous cavity (Figure 1(e)). The microforceps with closed tips was used to guide the ILM tissue to fall on the retinal surface in the vicinity of the hole followed by nudging the free ILM tissue into the hole on top of the inverted ILM tissue until they were securely in place (Figure 1(f)). Fluid-air exchange (FAX) up to the staphyloma margin or slightly above the detached retina level was performed for localized RD within the arcade. For RD extending to or beyond the equator, a drainage retinotomy was made with the vitreous cutter at the temporal upper detached retina for internal drainage and fluid-air exchange. No complete fluid-air exchange was intended as the air was only allowed to reach the margin of the staphyloma. Laser photocoagulation was done around the iatrogenic break. Finally, the air was replaced by 20% C_3_F_8_ infusion into the vitreous cavity. The patients were kept in a facedown position overnight and were allowed to take any position except supine for approximately one week. (Supplemental digital content of the video is available online at https://doi.org/10.1155/2017/3236516).

## 4. Results

**Figure 2:**
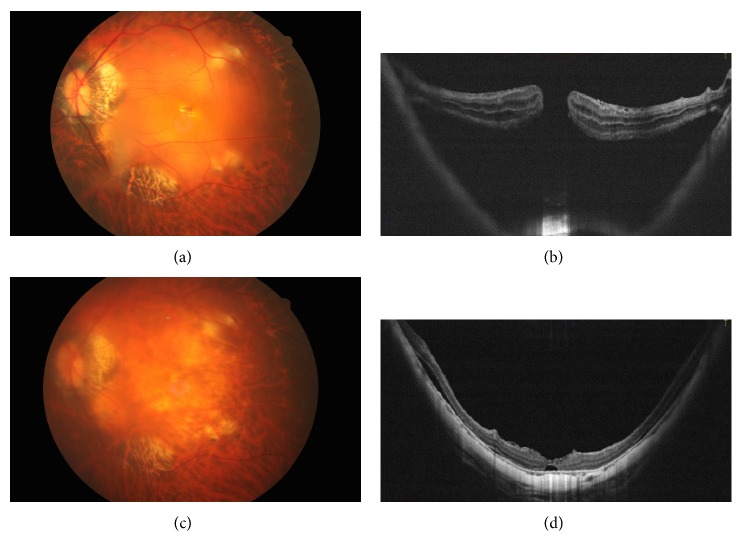
A 62-year-old woman developed a macular hole with retinal detachment in the left eye. Preoperative fundus photograph and optical coherence tomographic image showed the macular hole with localized posterior retinal detachment and adherent epiretinal membrane (a, b). Fundus photograph and optical coherence tomographic images 2 months after surgery showed the sealed macular hole with shallow residual subretinal fluid (c, d).

Nine consecutive patients underwent the above treatment. There were 3 men and 6 women. The average age was 64.55 ± 8.59 years old (ranging from 49 to 74 years). All cases had high myopia with an average axial length of 31.00 ± 1.25. All had reattached retina with sealed MH (Figure 2). The average visual acuity in LogMAR improved from 1.85 ± 1.07 preoperatively to 1.01 ± 0.42 postoperatively. The mean duration of the follow-up is 4.67 ± 1.00 months.  Table 1 summarizes the basic characteristics and surgical outcome of the cases. During surgery, 2 of the 9 cases had partial perifoveal ILM torn away along with the cortical vitreous or ERM during membrane-peeling procedures. Partial inverted ILM flap dislodgement was noted in these 2 eyes before Viscoat® and free ILM flap insertion. None showed any ILM flap position change after the insertion of free ILM flaps.

## 5. Discussion

Free ILM flap insertion had been used to treat chronic large MH or persistent MH after surgery in those cases with attached retina [9]. The technique is not suitable for MH with RD because there is no underlying structure to support the ILM flap and the inserted ILM tissue may either float up or sink down, unable to act as a bridging membrane. Inverted ILM flap, on the other hand, may suffer from ILM flipping back, thus losing the bridging effect. Recently, Lai et al. proposed a novel idea of using blood clot to stabilize and seal the ILM flap within the macular hole during air-fluid exchange [5]. A highly successful rate of hole closure was reported. However, drawing and introducing blood from outside the eye may carry the potential risk of infection. We recently proposed another technique by inserting sufficient amounts of ILM inside the hole to solve this problem [6]. But there are two major difficulties that leave rooms for improvement with this technique. The first issue is that there is still a natural tendency for the inverted flap to fold back, thus prolonging the insertion process. The repeated manipulation around or within the MH may cause tissue damage and possibly worsen the visual prognosis. The second issue is the possibility that parts of the ILM at the perifoveal area may sometimes be torn away along with the cortical vitreous above or ERM tissue before the ILM was exposed. This may result in insufficient ILM tissue inserted for a stable plugging effect, and the ILM may easily flip back even after repeated insertion. In this report we applied the inverted ILM flap insertion technique but modified the technique with two additional steps: first, using viscoelastic substance within the macular hole and on top of the ILM plug; second, using another piece of free ILM flap to plug the hole. Our preliminary results showed that our technique greatly stabilize the ILM in position, especially in the eyes that had insufficient perifoveal ILM left, which in turn facilitate the hole closure and retinal reattachment.

Viscoelastic substance was used before ILM peeling to prevent ICG dye entering into the hole and to decrease the ICG-stained area around the macular hole. A small amount of Viscoat injected would suffice. The viscoelastic substance was again used after inverted flap insertion. This extra procedure may serve two purposes: the first was to temporally cover the inserted ILM to lessen the possibility of ILM tissue flipping back; the second was to retard the mobility of the free ILM flap during insertion, making microforceps-guided free ILM insertion easier to perform. Because of the presence of the inverted ILM within the hole, the free ILM flap would not sink and lose into the subretinal space; because of the trapped free ILM flap, the underlying inverted ILM flap would be prevented from folding back and slipping out of the hole. These two tissues supported as well as checked each other, making the inserted ILM very stable. One of our cases with subretinal hemorrhage developed posterior pole bullous detachment during FAX because the blood clot temporally obstructed the iatrogenic temporal upper drainage site while the infused air pushed the peripheral subretinal fluid to the posterior subretinal space. Even in this extreme condition, the flap remained within the hole, indicating that this technique can make the inserted flap secure enough to withstand the significant turbulence during surgery. This additional procedure reduced the time for prolonged manipulation to fight against the flipping back tendency of the inserted flap; it also added sufficient ILM tissue to overcome the parafoveal ILM tissue loss during ERM removal.

## 6. Conclusion

The double ILM insertion technique may be a favorable option to secure the inserted ILM flaps, especially in the eyes with insufficient perifoveal ILM tissue. Further studies with a large case number and a control group should be done to confirm our observation.

## Supplementary Material

Supplementary digital content: The video shows the double ILM insertion technique. After the insertion of the inverted ILM into the macular hole, a free piece of ILM was nudged into the hole to further secure the inverted ILM in place

## Figures and Tables

**Table 1:** Demographic data of patients.

Number/age/sex/eye	Axial length	Extent of RD	Best-corrected visual acuity in LogMAR		Duration of follow-up (months)	Macular hole sealed
			Initial	Final		
1/63/F/OS	31.38	1	3.00	1.4	5	Yes
2/62/F/OS	30.86	1	1.30	1.00	6	Yes
3/74/F/OS	30.21	1	1.30	0.70	5	Yes
4/49/F/OD	29.31	1	0.69	0.66	4	Yes
5/67/M/OD	33.72	1	4.00	1.3	4	Yes
6/57/M/OS	31.58	1	0.82	0.22	5	Yes
7/67/M/OD	30.58	1	1.52	1.52	3	Yes
8/68/F/OD	30.11	2	2.00	1.30	6	Yes
9/64/F/OS	31.24	2	2.00	1.0	4	Yes

Retinal detachment extends to the equator or beyond. The extent of RD is 1. The localized retinal detachment is 2; F: female; M: male; OD: right eye; OS: left eye; LogMAR: logarithm of minimal angle of resolution.
